# Host Recovery and Reduced Virus Level in the Upper Leaves after *Potato virus Y* Infection Occur in Tobacco and Tomato but not in Potato Plants

**DOI:** 10.3390/v7020680

**Published:** 2015-02-11

**Authors:** Xianzhou Nie, Teresa A. Molen

**Affiliations:** Potato Research Centre, Agriculture and Agri-Food Canada, P.O. Box 20280, 850 Lincoln Road, Fredericton, NB E3B 4Z7, Canada; E-Mail: teresa.molen@agr.gc.ca

**Keywords:** PVY, host recovery, virus-derived small RNA

## Abstract

In this study, the recovery phenomenon following infection with *Potato virus Y* (PVY) was investigated in tobacco (*Nicotiana tobaccum*), tomato (*Solanum lycopersicum*) and potato (*Solanum tuberosum*) plants. In tobacco plants, infection of severe strains of PVY (PVY^N^ or PVY^N:O^) induced conspicuous vein clearing and leaf deformation in the first three leaves above the inoculated leaves, but much milder symptoms in the upper leaves. The recovery phenotype was not obvious in tobacco plants infected with PVY strain that induce mild symptoms (PVY^O^). However, regardless of the virus strains, reduction in PVY RNA levels was similarly observed in the upper leaves of these plants. Removal of the first three leaves above the inoculated leaves interfered with the occurrence of recovery, suggesting that the signal(s) mediating the recovery is likely generated in these leaves. In PVY^N^ or PVY^N:O^ but not in PVY^O^-infected tobacco plants, the expression of PR-1a transcripts were correlated with the accumulation level of PVY RNA. Reduced level of PVY RNA in the upper leaves was also observed in infected tomato plants, whereas such phenomenon was not observed in potato plants. PVY-derived small RNAs were detected in both tobacco and potato plants and their accumulation levels were correlated with PVY RNA levels. Our results demonstrate that the recovery phenotype following PVY infection is host-specific and not necessarily associated with the expression of PR-1a and generation of PVY small RNAs.

## 1. Introduction

Multiple strategies are employed by plants to cope with virus invasion. These strategies include the inherited and pathogen-specific resistance conferred by a resistance (*R*) gene [1], the induced and pathogen-nonspecific resistance brought about by systemic acquired resistance (SAR) [2,3,4,5], and the pathogen-derived and pathogen-specific resistance mediated by RNA silencing [4]. The *R* gene-mediated resistance is the classical mode of resistance in host plants against incompatible pathogens including viruses [1], falling into the gene-for-gene model [6]. The SAR, on the other hand, is a basal defense mechanism mediated by signal molecules such as salicylic acid (SA), ethylene and jasmonic acid against a broad spectrum of pathogens [2]. The SAR is normally coupled with the induction of various pathogenesis-related (PR) proteins (e.g., PR1) and stress-related proteins (e.g., Hsp90) as well as proteins involved in synthesis of the signal molecules (e.g., ACC oxidase) [2,3,4,5]. The RNA silencing-directed resistance is mediated by small interfering RNAs (siRNA) of ~21–26 nucleotides (nt) in length [7,8,9], generated through the cleavage of a double-stranded, or an imperfect stem-loop, RNA molecule by a Dicer enzyme [10].

Host recovery, a phenomenon that is characterized by an initial severe symptom expression upon viral infection followed by a reduced symptom severity in newly emerging leaves [11], has been reported in many plant species [5,11,12]. Studies have demonstrated that RNA silencing plays a role in host recovery in different plant species against both RNA and DNA viruses [12,13,14,15].

*Potato virus Y* (PVY), genus *Potyvirus*, family *Potyviridae*, is a single-stranded positive-sense RNA virus that has a broad host range and induces severe symptoms in many plant species including potato and tobacco plants [16,17,18]. Multiple strains and sub-strains including the common strain (PVY°), the tobacco veinal necrosis strain (PVY^N^), the potato tuber necrosis sub-strain (PVY^NTN^) and the N-O strain (PVY^N:O^) exist. It is noteworthy that PVY^N:O^ and the recombinant PVY^NTN^ isolates (*i.e.*, European-PVY^NTN^) possess a hybrid genome comprising segments of PVY^O^ and PVY^N^ [17,19]. Like PVY^N^ and PVY^NTN^, most PVY^N:O^ isolates induce vein clearing, veinal necrosis, foliar deformation and stem necrosis in tobacco plants [5,19,20]. However, exception occurs. For instance, among the PVY^N:O^ isolates collected in Manitoba, Canada, three distinct subtypes, namely the mild, the intermediate and the severe, were recognized based on their pathogenicity on tobacco plants [19]. Using a severe isolate of PVY^N:O^ and tobacco plants as a model system, the interactions between PVY^N:O^ and tobacco were characterized [5]. Three stages, namely the virus incubation stage (0–7 dpi), the rapid symptom-progress stage (8–14 dpi) and the partial recovery stage (≥15 dpi), have been recognized. Salicylic acid suppresses the symptom severity and virus level, but does not affect the symptom development and the recovery phenotype [5], suggesting that SAR does not play a role in recovery.

In this study, the recovery phenotype was further investigated in tobacco upon infection with various strains of PVY. The results demonstrate that recovery and reduced virus level in the upper leaves took place in tobacco, regardless of virus isolates/strains and the initial symptoms. Using the virus level as an indicator, the recovery was revealed to occur in tomato but not in potato plants, demonstrating that the recovery phenotype is host-dependent. Despite of differences, PVY genome-derived small RNAs of ~21 nt were detected in both tobacco and potato plants.

## 2. Results

### 2.1. Reduction in PVY Symptom Severities is Associated with Lower Viral RNA Level

Previous studies indicate that tobacco plants infected with a severe isolate of PVY^N:O^, PVY^N:O^-Mb58, undergoes three stages, *i.e.*, incubation, rapid symptom progressing and partial recovery stages [5].

**Figure 1:**
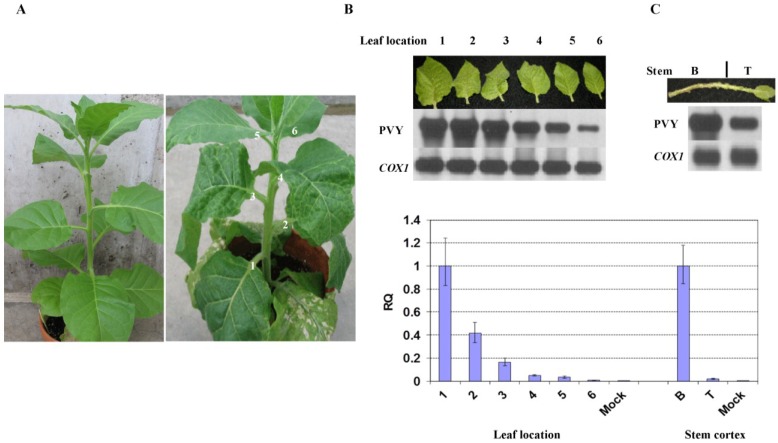
Recovery phenotype in tobacco plant after infection with PVY^N:O^-Mb58. (**A**) Symptoms in *Potato virus Y* (PVY)-infected plant (right) at 14 days post-inoculation (dpi). Left, mock-inoculated plant; (**B**,**C**) RT-PCR-Southern blot analysis of PVY level and *COX1* in +1 to +6 leaves as well as stem cortex at the bottom (B) and top (T); (**D**) Real-time RT-PCR analysis of the relative PVY level (RQ) after normalization with the *COX1* level in leaves and stem segments.

To further investigate whether the attenuated symptoms in the upper leaves and stems are associated with virus level, stem segments and leaves that emerged after inoculation and were physically located +1 to +6 nodes above the inoculated leaves were analyzed for the relative virus amounts. At 14 days post-inoculation (dpi), +1 to +3 leaves demonstrated severe vein clearing and malformation whereas the above leaves exhibited much milder symptoms, indicating recovery (Figure 1A). Reverse transcription-polymerase chain reaction (RT-PCR) followed by Southern blot analysis revealed higher levels of PVY in the first three leaves and lower levels in the upper leaves (Figure 1B). Further analysis revealed a similar pattern in the stem: the lower segment showed severe necrosis and contained higher levels of PVY; whereas the upper segment exhibited little or no visible necrosis and had much lower levels of PVY (Figure 1C). Similar levels of cytochrome c oxidase subunit I gene (*COX1*), a housekeeping gene served as a reference [5], were found in both segments. Real-time quantitative RT-PCR (qRT-PCR) with *COX1* as the internal reference produced the same results (Figure 1D).

**Figure 2:**
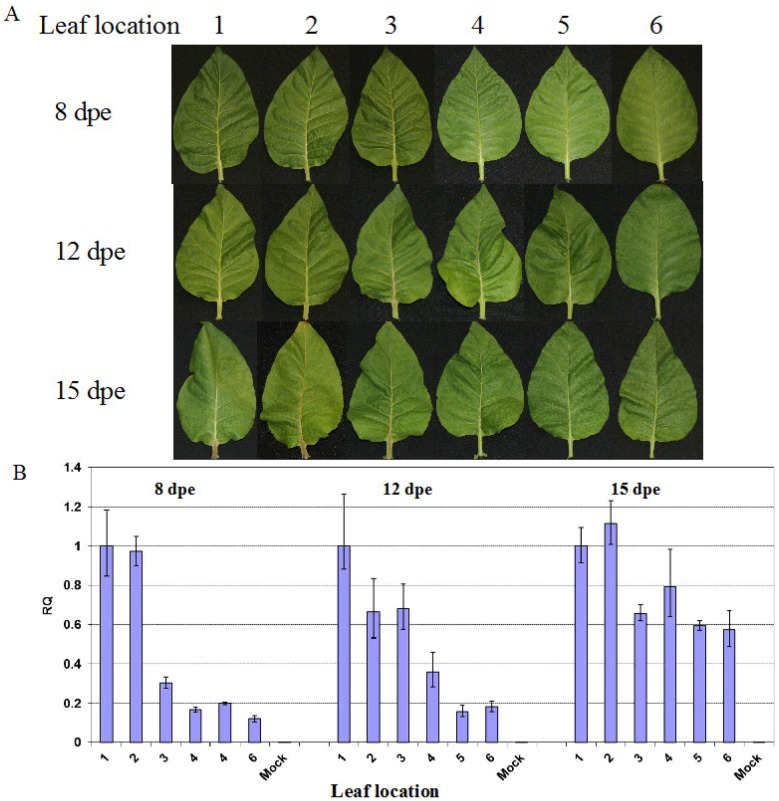
Effects of leaf age on the recovery phenotype in latterly emerging leaves of tobacco plants infected with PVY^N:O^-Mb58. (**A**) Symptoms in +1 to +6 leaves at the leaf age of 8, 12 and 15 days post-emergence (dpe); (**B**) Real-time RT-PCR analysis of the relative PVY level (RQ) after normalization with the *COX1* level in the leaves.

To investigate whether the decreased levels of PVY^N:O^-Mb58 in the more recovered leaves were due to the leaf age, leaves at +1 to +6 node position were analyzed at the leaf age of 8, 12 and 15 days post-emergence (dpe). Vein necrosis was visible in the +1 to +3 leaves (Figure 2A), and as leaves aged, the symptoms became more severe. Whereas in the upper leaves, no obvious necrotic symptoms were observed; and as leaf aged, chlorosis became prominent. Real-time RT-PCR was carried out to analyze PVY level in the leaves.

At 8 dpe, a relative high concentration was found in the +1 and +2 leaves, and as leaf position increased, lower levels of PVY were found in the leaves (Figure 2B, 8 dpe). A similar trend was found in leaves at both 12 and 15 dpe even though the differences of PVY titre among the lower and upper leaves became much less prominent at 15 dpe (Figure 2B, 15 dpe). These results suggest that the attenuated symptoms in the more latterly emerging leaves are likely associated with PVY level, and the recovery phenotype is virtually reflected in both reduced virus accumulation and attenuated symptoms in the upper leaves. The lower difference in virus level between the upper and the lower leaves at 15 dpe, when the lower leaves show signs of senescence, suggest that the virus concentration is growing at different paces. In lower leaves, the virus reaches its saturation level rapidly whereas in upper leaves, the virus accumulates at a much slower pace.

### 2.2. Reduced Virus Level in Upper Leaves Occurs in Tobacco upon Infection with Various PVY Strains

**Figure 3:**
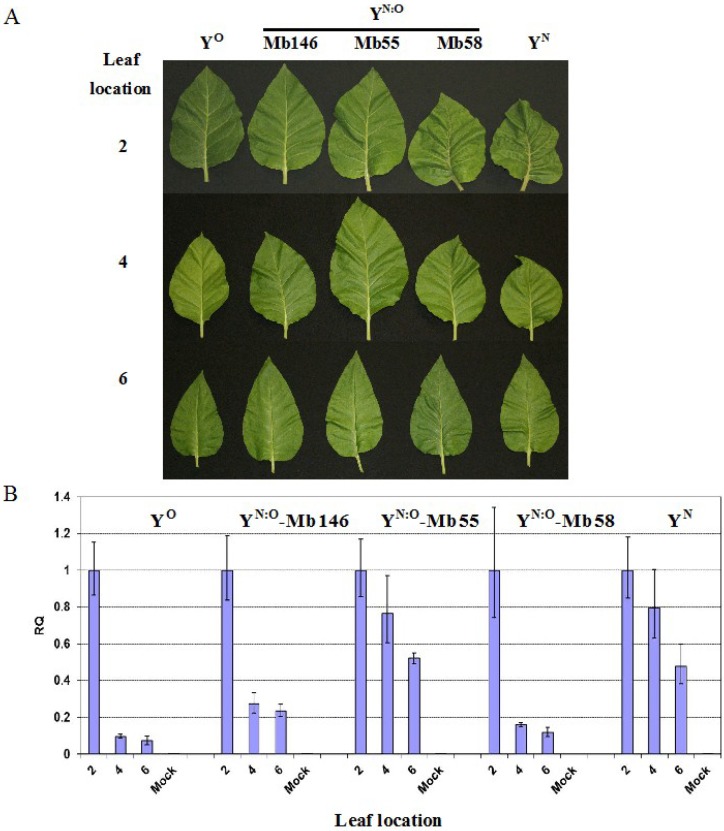
Symptoms and virus level in leaves of tobacco plants infected with different strains/isolates of PVY. (**A**) Symptoms in +2, +4 and +6 leaves of plants infected with PVY^O^ (Y^O^, isolate RB), PVY^N:O^ (Y^N:O^, isolates MB146, Mb55 and Mb58) and PVY^N^ (Y^N^, isolate N-Jg) at the leaf age of 12 days post-emergence; (**B**) Relative PVY level (RQ) in leaves. The virus level was obtained using real-time RT-PCR analysis of PVY upon normalization with *COX1* level in the leaves.

Unlike PVY^N^ and PVY^N^-like isolates such as PVY^N:O^ and PVY^NTN^, PVY^O^ does not induce veinal and stem necrosis in tobacco [19,20,21,22,23]. Because mosaic is the predominant symptom in PVY^O^-infected plants, recovery in terms of symptom attenuation in the plants is not as obvious as that in those infected with necrosis-inducible strains. To reveal whether recovery reflected in reduced virus accumulation in upper leaves is a common reaction in tobacco to all strains of PVY, qRT-PCR was performed in +2, +4 and +6 leaves at 12 dpe (Figure 3B). In plants inoculated with PVY^O^ and PVY^N:O^-Mb146, a decrease of virus concentration was found in leaves located from positions +2 to +6 (Figure 3B) even though no significant attenuation of symptoms was found in the more latterly emerged leaves (Figure 3A). In plants infected with PVY^N:O^-Mb55, PVY^N:O^-Mb58 and PVY^N^, a clear trend of symptom-severity attenuation based on veinal and petiole necrosis occurred in the more latterly emerged leaves (Figure 3A). Correlated with the weaker symptoms in the upper leaves, especially the +6 leaves, a lower level of virus was found (Figure 3B).

### 2.3. Removal of the Lower Leaves Affects the Occurrence of Recovery in Upper Leaves

**Figure 4:**
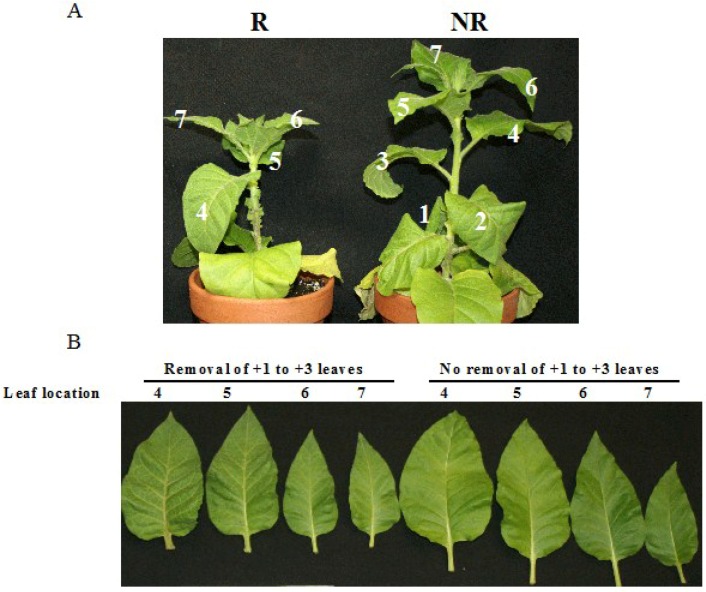

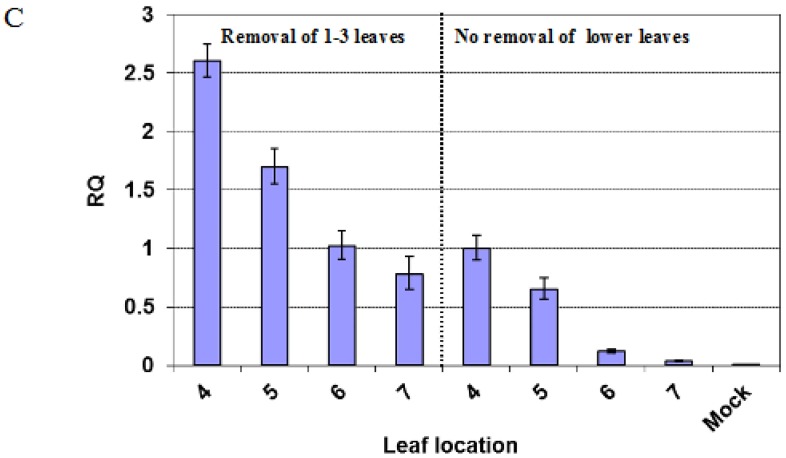
Effects of removal of earlier leaves on symptoms and virus level in latter leaves of tobacco plants infected with PVY^N:O^-Mb58. (**A**) Symptoms in plants with (R) and without (NR) the removal of the +1 to +3 leaves; (**B**) Leaves at +4 to +7 node position at the leaf age of 12 days post-emergence (dpe) from plants with and without the removal of the leaves at +1 to +3 node position; (**C**) Relative PVY level (RQ) in leaves. The virus level was obtained using real-time RT-PCR analysis of PVY upon normalization with the *COX1* level in the leaves.

To investigate whether virus accumulation in lower leaves contribute to the occurrence of recovery in upper leaves, +1 to +3 leaves were removed when they became emerged (*i.e.*, 0 dpe) in plants inoculated with PVY^N:O^-Mb58. The plants were smaller in size in comparison to those whose leaves were not removed (Figure 4A). However, the development of the plants, especially leaf numbers and emerging time, was not noticeably affected. Of the +4 to +7 leaves studied at 12 dpe, the +4 and +5 leaves of the plant whose +1 to +3 leaves were removed exhibited more severe veinal and petiole necrosis than their counterparts in plant without leaf-removal (Figure 4B). When the leaves from the same plants were compared, a gradual veinal/petiole necrosis-attenuation was found in leaves from position +4 to +7 in the plant with leaf removal; however, since the lower leaves (e.g., +4 and +5 leaves) in the plant without leaf removal already “recovered”, no further necrosis attenuation was recorded in the further upper leaves (+6 and +7). A significant higher level of virus was detected in the leaves from plant with leaf removal (Figure 4C). When the leaves from the same plant were compared, a gradual decrease of virus level was found in the leaves as their locations increased in both plants (Figure 4C). These results suggest that the signal(s) leading to recovery might have been generated in the earlier emerged leaves; removing these leaves would delay the overall recovery.

### 2.4. Recovery is Independent from PR-1a Expression

**Figure 5:**
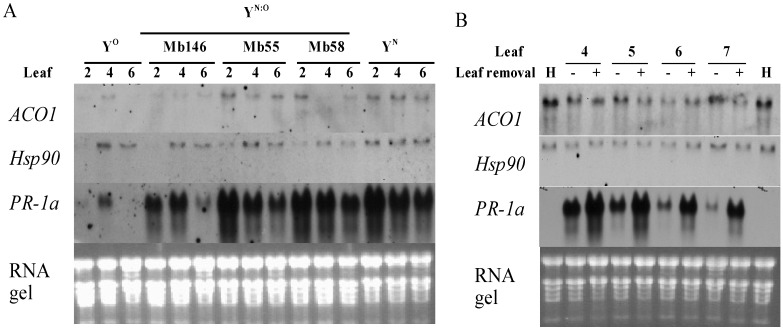
Northern blot analysis of the expression of *ACO1*, *Hsp90* and *PR-1a* in leaves emerged after inoculation in plants infected with different strains/isolates of PVY. (**A**) Gene expression in +2, +4 and +6 leaves of plants infected with PVY^O^ (Y^O^, isolate RB), PVY^N:O^ (Y^N:O^, isolates MB146, Mb55 and Mb58) and PVY^N^ (Y^N^, isolate N-Jg) at the leaf age of 12 days post-emergence (dpe); (**B**) Gene expression in +4 to +7 leaves in plants with (+) and without (−) the removal of the +1 to +3 leaves. Northern blots were performed using total RNA extracted from the pooled leaves of 4 plants. *ACO1*, ACC oxidase 1; *Hsp90*, heat-shock protein 90; *PR-1a*, pathogenesis-related protein 1a; H, leaves from healthy plants. The experiments were repeated two times, and similar results were obtained.

The expression of *PR-1a*, *Hsp90* and ACC oxidase 1 (*ACO1*) was investigated to reveal whether the genes are involved in recovery in tobacco. In plants infected with PVY^N:O^-Mb55, PVY^N:O^-Mb58 and PVY^N^, the highest expression of *PR-1a* was found in +2 leaf, followed by +4 and +6 leaves (Figure 5A), a pattern similar to PVY level in those plants (Figure 3B). However, in plants infected with PVY° and PVY^N:O^-Mb146, the highest and the lowest expression of *PR-1a* was found in the +4 and +6 leaves, respectively. When leaves at the same position but from different plants infected with different isolates of PVY were compared, lowest expression was found in PVY^O^, followed by PVY^N:O^-Mb146, PVY^N:O^-Mb55, PVY^N:O^-Mb58 and PVY^N^ (Figure 5A). In PVY^N:O^-Mb58 infected plants, removal of leaves +1 to +3 led to higher levels of *PR-1a* transcript in the +4 to +7 leaves than their counterparts (Figure 5B). Together, the results indicate that *PR-1a* expression is closely associated with symptom severity other than recovery *per se*.

*Hsp90* expressed slightly more in +4 leaf than in +2 and +6 leaves in all PVY-infected plants (Figure 5B). However, no obvious difference was found between the PVY^N:O^-Mb58 infected plants whose +1 to +3 leaves were removed or kept (Figure 5B). A slightly higher expression of *ACO1* was found in leaves of plants infected with PVY^N:O^-Mb55, PVY^N:O^-Mb58 and PVY^N^ than in leaves of PVY^O^ and PVY^N:O^-Mb146 (Figure 5A). In PVY^N:O^-Mb58-infected plants, except in +6 leaf, *ACO1* was expressed slightly higher in the leaves of plants whose +1 to +3 leaves were removed (Figure 5B).

### 2.5. Reduced Virus Level in Upper Leaves after PVY Infection Occurs in Tomato but not in Potato Plants

**Figure 6:**
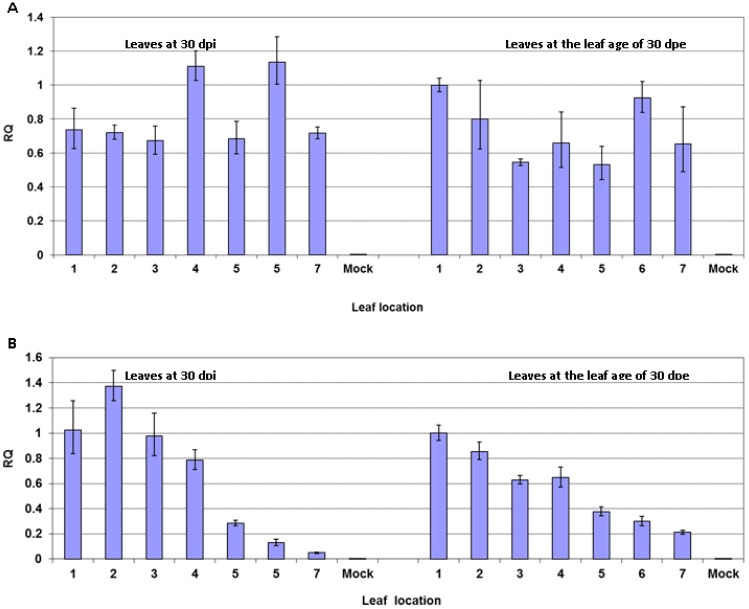
Virus level in leaves emerged after inoculation in potato and tomato plants infected with PVY^N:O^-Mb58. (**A**) Leaves of potato (cv. “Ranger Russet”) at 30 days post-inoculation (dpi) and at the leaf age of 30 days post-emergence (dpe); (**B**) Leaves of tomato (cv. “Sheyenne”) at 30 dpi and at the leaf age of 30 dpe. The relative virus level (RQ) was obtained by real-time RT-PCR analysis of PVY RNA upon normalization with *COX1* mRNA in leaves.

To investigate whether PVY infection also induces recovery in other solanaceous plants, potato (cv. “Ranger Russet”) and tomato (cv. “Sheyenne”) were inoculated with PVY^N:O^-Mb58. Both “Ranger Russet” potato and “Sheyenne” tomato are susceptible to the isolate. In potato plants, PVY^N:O^ induced roughness and mottling in all leaves emerged after inoculation, regardless of their physical location. No obvious symptoms were observed in tomato plants upon virus infection. In potato, qRT-PCR analysis demonstrated a similar level of viral RNA in all leaves at 30 days post-inoculation or at 30 days post-emergence (Figure 6A), ~10 days prior to the appearance of leaf senescing. Whereas, in tomato, the highest levels of viral RNA were found in +1 to +4 leaves at both 30 dpi and 30 dpe (Figure 6B), ~10 days prior to the appearance of leaf senescing.

### 2.6. PVY-Derived siRNAs are Generated in both Tobacco and Potato Plants

**Figure 7:**
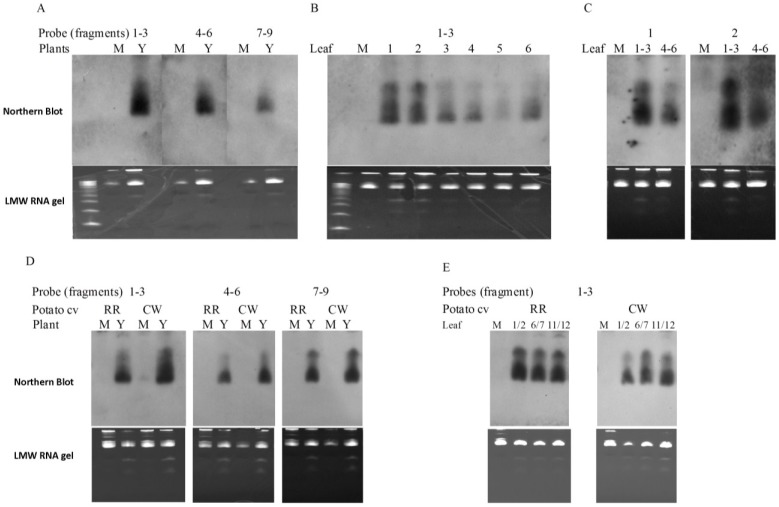
Northern blot detection of PVY-derived small RNA (siRNA) in leaves of tobacco and potato plants infected with PVY^N:O^-Mb58. (**A**) Detection of PVY-siRNA in leaves (12 dpe) of PVY (Y)- and mock (M)-inoculated tobacco plants using fragments covering various segments of the complete PVY genome; (**B**) Detection of PVY-siRNA in leaves of PVY-inoculated potato plants using probes of PVY fragments 1–3; (**C**) Detection of PVY-siRNA in different leaves of PVY-inoculated tobacco plants using probes of PVY fragment 1; (**D**) Detection of PVY-siRNA in leaves (30 dpe) of PVY- and mock-inoculated potato plants using fragments covering various segments of the complete PVY genome; (**E**) Detection of PVY-siRNA in various leaves of PVY-inoculated potato plants using probes of PVY fragments 1–3. The PVY fragments 1–9 cover the nucleotide segment of PVY^N:O^ genome 5'end–1200, 1100–2400, 2300–3500, 3400–4600, 4500–5700, 5600–6800, 6700–7900, 7800–9000, 8900–3'end, respectively. The far left lane in low-molecular-weight (LMW) gels is 10 bp DNA step ladder (Promega. Bottom to top: 10 bp to 100 bp in exactly 10 bp increments). M, mock; Y, PVY; RR, potato cv. “Ranger Russet”; CW, potato cv. “Cal White”.

RNA silencing has been linked to recovery in a number of plant species infected with different viruses [12,13,14,24]. To investigate whether short RNAs originated from PVY were also produced in PVY-infected plants, and moreover, whether siRNA played a role in the apparent recovery in tobacco and the non-recovery in potato, Northern blot analysis of siRNA with probes derived from various sections of PVY genome was carried out. As shown in Figure 7A, short RNAs, which were estimated to be ~21–24 bp based on the 10 bp DNA step ladder and were confirmed to be 21 bp by deep-sequencing [25], were detected in leaves of PVY^N:O^-Mb58-infected tobacco plants by probes originated from various sections (fragments 1–3: nt 1–~3300; fragments 4–6: ~nt 3250–~nt. 6500; fragments 7–9: ~nt 6400–3' end) of the virus genome. These results demonstrated that the PVY-siRNAs were a population of siRNAs that might have been produced from all three sections (*i.e.*, 5'end to ~nt 3300; ~3200–~ 6500; and nt 6400 to 3'end) of PVY genome. Using the probes covering the 5' proximal section (*i.e.*, 5'end to ~nt. 3300) of PVY genome, siRNAs were detected in all leaves locating from +1 to +6 above the inoculated leaves (Figure 7B, +1 to +6). Moreover, as leaf position increased, siRNA levels decreased (Figure 7B, +1 to +5) even though the +6 leaf exhibited a higher level of siRNA than the +5 leaf did (Figure 7B, +5 and +6).

Further experiments were carried out on potato plants upon infection with PVY^N:O^-Mb58. Two potato cultivars, “Ranger Russet” (RR) and “Cal White” (CW), both susceptible to the PVY isolate, were used. RR showed typical mosaic symptoms in leaves 3 weeks post-inoculation whereas CW did not show obvious symptoms (data not shown), consistent with the varietal descriptions and previous reports [21,22,26]. Small RNAs were detected from leaves of both PVY-infected cultivars by all three PVY probes (Figure 7C). Further analysis using the 5'-end probe (*i.e.*, probe for 5'end–~ nt 3300) showed that, in RR, a similar level of PVY-small RNAs was present in all leaves emerged after inoculation, regardless of their locations (Figure 7D, RR, 1/2, 6/7, and 11/12); and in CW, leaves 11/12 appeared to have the highest levels of PVY-siRNA, followed by leaves 6/7, and leaves 1/2 (Figure 7D, CW, 1/2, 6/7, and 11/12).

## 3. Discussion

Host recovery, which is manifested in symptom attenuation in the upper part of plants after viral infection, occurs in many plant species with different viruses [12,13,14,24]. Previously, we demonstrated the existence of host recovery in tobacco plants upon PVY^N:O^ infection [5]. In this study, the recovery in tobacco, potato and tomato in response to different strains of PVY is further examined. Our results confirmed a reduction of the viral RNA level during the recovery process in tobacco, consistent with the attenuated symptom severity in the plants. In tomato, although no visual recovery was observed, reduced virus level was detected at the upper leaves after PVY-infection, indicating the occurrence of host recovery in these plants. Interestingly, no host recovery was detected in potato as PVY-infected plants did not exhibit attenuated symptom severity nor did the plants exhibit reduced viral RNA level in upper leaves. Together, the results suggest that (1) reduced virus level is a more accurate indicator than the attenuated symptom severity for indication of a host recovery; (2) host recovery occurrence appears to be host-specific. This is consistent with a general observation that potato plants exhibit more severe symptoms in secondary infections (tuber-borne infections) than primary infections (current season infections) [22].

Signals leading to recovery are likely originated from lower leaves (the source) as removal of these leaves delayed recovery in the upper and more latterly emerged leaves (the destination). The signals are unlikely associated with SAR as salicylic acid does not alter the recovery pattern in PVY^N:O^-infected tobacco plants [5]. Moreover, the expression of the pathogenesis related protein gene *PR-1a*, which has been shown to be induced in SAR against PVY^N:O^-Mb58 [5] but does not appear to play a role in virus resistance [27], is disassociated with the reduced virus level in upper leaves in plants infected with PVY^O^ and PVY^N:O^-Mb146. Similarly, the more elevated expression of *ACO1* in PVY^N:O^-Mb55, PVY^N:O^-Mb58 and PVY^N^ infected plants than that in PVY^O^ and PVY^N:O^-Mb146 infected plants suggest that ACO, a key enzyme involved in the synthesis of the phytohormone ethylene [28], is likely to be resulted from the severe necrosis induced by PVY^N:O^-Mb55, PVY^N:O^-Mb58 and PVY^N^. Ethylene is therefore unlikely involved in host recovery in tobacco upon PVY infection. Heat shock protein 90 (Hsp90) has been implicated to be involved in disease resistance in plants. However, the expression of *Hsp90* in different leaves of tobacco plants infected with different isolates of PVY does not appear to have a clear pattern, suggesting that *Hsp90* may not be involved directly in host recovery after PVY infection. 

Virus derived small interfering RNAs have been shown to work as silencing signals to specifically target the viral RNAs [9,29,30,31,32,33], leading to host recovery [12,24,34]. For RNA viruses in plants, the silencing starts with the biogenesis of siRNA from a double stranded RNA precursor during the virus infection/replication by host Dicer-like (DCL) proteins [35]. Depending on the DCL proteins, 21, 22 and 24 nucleotide siRNAs could be produced [36]. One strand of the siRNAs is selected and incorporated into RNA-induced silencing complexes (RISC), and the catalytically active RNase known as Argonaute (AGO) proteins within the complexes directs specific degradation of the complementary RNA molecules [37]. RNA silencing can spread from the site of viral infection to more distant tissues, a phenomenon termed systemic acquired silencing (SAS) [38].

Despite the fact that a cross-talk between SA-induced SAR and local RNA silencing in tobacco against *Tomato ringspot virus* and *Plum pox virus* has been indicated [14,28], SA appears to play a minimal role in host recovery against PVY as SA application does not alter the recovery phenotype and recovery timeline [15]. It is, therefore, reasonable to believe that other factors such as RNA silencing play a major role in recovery in the host plant. To examine this hypothesis, Northern blot was carried out to detect virus-derived small RNAs in the signal producing “source” leaves and the upper “recovered” leaves. It was initially hoped that siRNA leading to host recovery could be mapped to a specific segment(s) of the PVY RNA genome, as shown in geminivirus in cassava [12] and in Cymbidium ringspot tombusvirus in *N. benthamiana* [34]. However, using PVY^N:O^-Mb58 as a representative isolate, siRNAs corresponding to the complete genome were detected in the pooled leaves comprised of both lower “source” and upper “recovered” leaves, consistent with deep-sequencing results of siRNA from the PVY-infected tobacco plants obtained in a preliminary study [25]. Similarly, a continuous coverage of every genomic position of different positive RNA viruses by the virus derived small RNAs have been reported in different plants [39,40,41,42,43]. Whether all the siRNAs can work as “translocatable” silencing signals or only certain species of siRNA derived from a specific genome segment work as the signals or whether a minimal concentration of some specific siRNAs is need in order to trigger a defense reaction remain unknown. The gradual level decrease of siRNAs corresponding to the genome segment 5'end–~3500 nt from lower to upper leaves was coincident with that of viral RNA, suggesting a positive relationship between the virus level and siRNA level in the leaves. A similar trend was observed in potato that did not exhibit host recovery. Together, these results demonstrate that viral siRNA production is a common process in infected plants; and depending on host species, the presence of siRNA does not necessarily lead to host recovery.

## 4. Materials and Methods 

### 4.1. Virus Cultures and Plant Materials

*Potato virus Y* isolates PVY^N:O^-Mb55, PVY^N:O^-Mb58 and PVY^N:O^-Mb146 [37], PVY^O^-RB and PVY^N^-Jg [44], and virus-free plants of tobacco (*Nicotiana tobaccum*) cv. “Samsun”, tomato (*Solanum lycopersicum*) cv. “Sheyenne” and potato (*Solanum tuberosum*) cvs. “Ranger Russet” and “Cal White” were used in this study. All the materials are susceptible to PVY infection. Virus inoculums were maintained in tobacco cv. “Samsun”. They were periodically checked for their purity using RT-PCR and symptom characterization [19,44,45].

### 4.2. Plant Inoculation and Symptom Observations

Virus-free seedlings of tobacco, tomato and potato were planted in 5-inch-pots containing the premix, and kept in the greenhouse with a light/dark cycle of 16/8 h at 18 °C (D)–24 °C (L). The humidity in the greenhouse was 50%–70%, and the sunlight was supplemented with artificial light (~90 μmole·m^−2^·s^−1^). The maximum light intensity was 350 μmole·m^−2^·s^−1^. At 5-leaf-stage, the plants were inoculated mechanically with various isolates of PVY as described previously [5]. Briefly, 15 g leaves of virus culture plants were homogenized in 50 mL inoculation buffer (10 mM sodium phosphate buffer, pH 7.5, with 32 mM Na_2_SO_3_), and filtered through a double-layer cheese cloth, resulting in approximately 70 mL virus extract. Thereafter, top three leaves of virus-free seedlings were dusted with Carborundum and gently rubbed with a pestle in the presence of virus extract at a rate of 1.0 mL per plant. The plants were then maintained in the greenhouse under the conditions as described above. The symptom expression was recorded for up to 24 days post-inoculation.

### 4.3. Northern Blot

Total RNA was isolated from 5 g of whole leaf tissue using the LiCl procedure as described by Mohapatra *et al.* [46]. Twenty micrograms of total RNA were denatured and electrophoresed on a 1.25% agarose gel, containing 2.2 M formaldehyde and a trace of ethidium bromide [47], and transferred with 20× saline sodium citrate (SSC) onto Hybond-N+ membranes (GE Healthcare, Waukesha, WI, USA) using VacuGene XL (GE Healthcare) at 50–55 mbar. After RNA fixation by UV crosslink, prehybridization (15 min) and hybridization (overnight) with appropriate probe were performed using Gene Images AlkPhos Direct Labelling and Detection System (GE Healthcare) as recommended by the manufacturer. After hybridization, the membrane was washed to remove the excess probe and incubated with the CDP-star chemiluminescent detection reagents (GE Healthcare). The chemiluminescent signals were detected by exposure of X-ray film (X-AR, Kodak, Rochester, NY, USA) at room temperature. After being stripped by boiling the filter in 0.1% SDS (w/v) for 5 min, the membrane was re-probed with appropriate probe, incubated with the CDP-star reagents and autoradiographed.

### 4.4. Probe Preparation

PCR fragment of the gene of interest was inserted into the pDrive vector (QIAGEN, Valencia, CA, USA), sequenced, and thereafter used as template for making probe with the Gene Images AlkPhos Direct Labelling and Detection System (GE Healthcare). For PVY probe, three fragments (inserts) including the P1 (nt 1–~1100), P3 (nt ~2100–~3300) and the CP (nt ~8500–9700) obtained from PVY^N:O^-Mb112 [19] pooled and labelled. Potato aminocylcopropane-1-carboxylic acid (ACC) oxidase gene 1 (*ACO1*) (accession number AF384820) [27] was used as a probe for the *ACO* gene. A 990 bp tobacco heat-shock protein 90 (*Hsp90*) (accession number GQ845021) amplified by 5'GTTTGACTGACAAGAGCAAGCT3' (sense) and 5'GGATCTTGTTCTGCTGCAA3' (antisense), and a tobacco *PR-1a* (accession number X06930) fragment of 760 bp amplified with primers 5'TCTCTTTTCACAATTGCCTTCA3' (sense) and 5'AGACCATCAACACATGATTCG3' (antisense) were used as probes for their corresponding genes after confirmation by cloning and sequencing.

### 4.5. Reverse Transcription-Polymerase Chain Reaction (RT-PCR) and Real-Time RT-PCR

Reverse transcription-polymerase chain reaction was also used to analyze PVY levels as described previously [5]. Briefly, 1 μg of total RNA in 2.5 μL was mixed with 1 μL (1 μg) of hexanucleotides (Roche Diagnostics, Indianapolis, IN, USA), incubated at 68 °C for 8 min, and chilled on ice for 3 min. Six-point-five μL of the RT mixture was added to provide a final concentration of 50 mM Tris-HCl, pH 8.3, 75 mM KCl, 10 mM DTT, 2.5 mM MgCl_2,_ 1.0 mM of each dNTP (dATP, dTTP, dCTP, and dGTP), 5 U RNasin Ribonuclease Inhibitor (Promega, Madison, WI, USA) and 100 U Moloney murine leukemia virus reverse transcriptase (Promega). Samples were incubated at 42 °C for 1 h, and 95 °C for 2 min. Thereafter, 40 μL H_2_O was added to the cDNA mix, and the resulting cDNA solution was used for semi-quantitative PCR analysis of PVY level and gene expression.

One-step real-time quantitative RT-PCR was performed using the Power SYBR^®^ Green RNA-to-C_T_™ 1-step kit (Applied Biosystems, Foster City, CA, USA) according to manufacturers’ instructions. The final volume of the reaction mixture was 20 µL, containing 50 ng total RNA and 100 nM each PVY primers (FpN 5'-(nt 2147) AACCATGATGGATCTGGCTACAA (nt 2196)-3' and RpN 5'-(nt 2253) TTCTAGGCAGTTCTGCATCATGAA (nt 2230)-3' for PVY^N:O^ and PVY^N^ isolates (reference isolate, PVY^N:O^-Mb112, accession number AY745491); FpO 5'-(nt 2173) TATGATGGATTTGGCGACCACTTGT (nt 2193)-3' and RpO 5'-(nt 2251) TAAACTAGGCAGCTCTGCATCATG (nt 2228)-3' for PVY^O^ isolate (reference isolate, PVY°-139, accession number U09509) [48] and *COX* primers (COX1-A8, 5'-GGTCGGACATACCTGAAAC-3', and COX1-AA8, 5'-CCAAAAGTATGAAAAGCTGGAG-5') [49]. The reaction included the reverse transcription at 48 °C for 30 min, the activation of AmpliTaq^®^Gold DNA Polymerase UP (Ultra Pure) (Applied Biosystems) at 95 °C for 10 min, and the PCR for 35 cycles, each with 15 s of denaturation at 95 °C and 1 min of annealing and extension at 60 °C. Fluorescence signals from each sample were recorded and analyzed using StepOne™ Software v2.1 (Applied Biosystems). Each pooled RNA sample was analyzed with three replicate reactions. The comparative C_T_ (ΔΔ C_T_) method was employed to analyze PVY levels on the StepOnePlus™ Real-Time PCR System (Applied Biosystems) [50]. Melt curves of the amplicons were performed after the RT-PCR in order to detect any nonspecific amplification. PCR efficiencies for both separately were found to be between 91%–100%.

### 4.6. RT-PCR-Southern Blot

Amplification products from RT-PCR (described previously) were fractionated by electrophoresis on 1.5% agarose gel containing 0.5 µg/mL ethidium bromide. After electrophoresis, the gel was blotted to Hybond-N+ (Amersham Biosciences, Piscataway, NJ, USA) according to the manufacturer’s instructions, and the resulting membrane was hybridized with probes corresponding to the gene of interest. Probes were generated through PCR amplification and used as template for labeling with the Gene Images AlkPhos Direct Labeling and Detection System (Amersham Biosciences) according to the manufacturer’s instructions. After hybridization, the membrane was washed to remove the excess probe and incubated with the CDP-star chemiluminescent detection reagents (Amersham Biosciences). The chemiluminescent signals were detected by exposure of X-ray film (X-AR, Kodak) at room temperature.

### 4.7. Isolation of Low Molecular Weight (LMW) RNA

Small molecular RNA was isolated from 1 g leaf tissue using the *mir*Vana™ miRNA Isolation kit (Ambion, Austin, TX, USA) following the manufacturer’s protocol with the addition of Plant RNA Isolation Aid (Ambion). Briefly, 1 g leaf tissue was powdered in liquid nitrogen and homogenized with 5 mL lysis/binding buffer and 0.5 mL plant isolation aid. 0.55 mL miRNA homogenate was added to the extract and incubated on ice for 10 min. After extraction with 1:1 (v/v) water-saturated phenol and chloroform: isoamyl alcohol (24:1, v/v), the aqueous phase was transferred to a new tube, and a 1/3 volume of 100% ethanol was added. The resulting mixture was passed through a Filter Cartridge containing a glass-fiber filter (Ambion), in which the high molecular weight RNA was retained, and the aliquots (~700 µL) containing the low molecular weight (LMW) RNA was collected in a filtrate. Two third volume of 100% ethanol was added to the filtrate and applied to a new Filter Cartridge. Finally, the LMW RNA was eluted by adding 100 µL of 95 °C nuclease-free water upon centrifugation in a microcentrifuge at 5000 rpm for 2 min. The resulting LMW RNA was quantified using a NanoDrop 2000 UV-Vis Spectrophotometer (Thermo Scientific, Wilmington, DE, USA).

### 4.8. Northern Blot Analysis of Small RNA

Three µg LMW RNA was separated in a 15% denaturing polyacrylamide (19:1) gel containing 8 M urea and 0.5× TBE. The gel was stained in 3× GelRed™ (Biotium, Hayward, CA, USA) in the presence of 0.1 M NaCl for 1.5 h, photographed with a FluorChem^TM^ Imaging System (Alpha Innotech, San Leandro, CA, USA), and rinsed in sterile water for 15 min followed by rinsing in 20× SSC for two times, each 15 min. The LMW RNA was transferred to Hybond™-N+ (GE Healthcare) overnight in 20× SSC via capillary. The membrane was rinsed briefly in 2× SSC followed by UV crosslinking at 150 ml with a GS Gene Linker™ UV Chamber (Bio-Rad Laboratories, Richmond, CA, USA). The 10 bp DNA step ladder (Promega) was used to in the gel electrophoresis.

Nine overlapping fragments (PVY1-PVY9) spanning the complete PVY^N:O^-Mb112 genome (accession number AY745491) were generated by RT-PCR using primers described previously [23] and cloned into pDrive cloning vector (Qiagen, Valencia, CA, USA). Upon confirmation by sequencing, the plasmids were linearized with *Bam*H1 and used to generate DIG-labelled RNA probes with the DIG RNA Labelling kit (Roche Diagnostics). The resulting RNA probes were quantified spectrophotometrically, and the size (~1.1 kb) and integrity were verified by denaturing RNA gel electrophoresis [47].

Hybridization was performed as described above. Briefly, the membranes were prehybridized in 20 mL DIG Easy Hyb (Roche Diagnostics) at 40 °C for 1 h, followed by hybridization with appropriate RNA probes (~1.2 µg/per probe) at 40 °C overnight. The membranes were then washed two times with 2× SSC/0.1% SDS at 50 °C, each for 15 min. Immunological detection using DIG Luminescent Detection Kit (Roche Diagnostics) was carried out following manufacturer’s guidelines. The membrane was finally exposed to Hyperfilm™ECL (GE Healthcare) and developed using GBX fixer and developer (Kodak). Sizes of the small RNAs were estimated based on the 10 bp DNA step ladder.

## 5. Conclusions

This research demonstrates that recovery took place in tobacco, regardless of virus isolates/strains and the initial symptoms. Using the virus level as an indicator, the recovery was revealed to occur in tomato but not in potato plants, demonstrating that the recovery phenotype is host-specific. Despite of differences, PVY genome-derived small RNAs of ~21 nt were detected in both tobacco and potato plants.

## References

[1] Kang B.C., Yeam I., Jahn M.M. (2005). Genetics of plant virus resistance. Ann. Rev. Phytopathol..

[2] Durrant W.E., Dong X. (2004). Systemic acquired resistance. Annu. Rev. Phytopathol..

[3] Gilliland A., Murphy A.M., Wong C.E., Carson R.A., Carr J.P., Tuzun S., Bent E. (2006). Mechanisms involved in induced resistance to plant viruses. Multigenic and Induced Systemic Resistance in Plants.

[4] Lewsey M.G., Carr J.P. (2009). Effects of DICER-like proteins 2, 3 and 4 on cucumber mosaic virus and tobacco mosaic virus infections in salicylic acid-treated plants. J. Gen. Virol..

[5] Nie X. (2006). Salicylic acid suppresses *Potato virus Y* isolate N:O-induced symptoms in tobacco plants. Phytopathology.

[6] Flor H.H. (1971). Current status of the gene-for-gene concept. Annu. Rev. Phytopathol..

[7] Hamilton A., Voinnet O., Chappell L., Baulcombe D. (2002). Two classes of short interfering RNA in RNA silencing. EMBO J..

[8] Kundu J.K., Briard P., Hily J.M., Ravelonandro M., Scorza R. (2008). Role of the 25–26 nt siRNA in the resistance of transgenic *Prunus domestica* graft inoculated with plum pox virus. Virus Genes.

[9] Ruiz-Ferrer V., Voinnet O. (2009). Roles of plant small RNAs in biotic stress responses. Annu. Rev. Plant Biol..

[10] Moazed D. (2009). Small RNAs in transcriptional gene silencing and genome defence. Nature.

[11] Wingard S.A. (1928). Hosts and symptoms of ring spot, a virus disease of plants. J. Agric. Res..

[12] Chellappan P., Vanitharani R., Fauquet C.M. (2004). Short interfering RNA accumulation correlates with host recovery in DNA virus-infected hosts, and gene silencing targets specific viral sequences. J. Virol..

[13] Jovel J., Walker M., Sanfaçon H. (2007). Recovery of *Nicotiana benthamiana* plants from a necrotic response induced by a nepovirus is associated with RNA silencing but not with reduced virus titer. J. Virol..

[14] Jovel J., Walker M., Sanfaçon H. (2011). Salicylic acid-dependent restriction of Tomato ringspot virus spread in tobacco is accompanied by a hypersensitive response, local RNA silencing, and moderate systemic resistance. Mol. Plant-Microbe Interact..

[15] Rodríguez-Negrete E.A., Carrillo-Tripp J., Rivera-Bustamante R.F. (2009). RNA silencing against geminivirus: Complementary action of posttranscriptional gene silencing and transcriptional gene silencing in host recovery. J. Virol..

[16] Gray S., de Boer S., Lorenzen J., Karasev A., Whitworth J., Nolte P., Singh R., Boucher A., Xu H. (2010). *Potato virus Y*: An evolving concern for potato crops in the United States and Canada. Plant Dis..

[17] Karasev A.V., Gray S.M. (2013). Continuous and emerging challenges of *Potato virus Y* in potato. Annu. Rev. Phytopathol..

[18] Nie X., Singh M., Pelletier Y., McLaren D. (2013). Recent advances on *Potato virus Y* research in Canada. Am. J. Potato Res..

[19] Nie X., Singh R.P., Singh M. (2004). Molecular and pathological characterization of N:O isolates of the *Potato virus Y* from Manitoba, Canada. Can. J. Plant Pathol..

[20] Hu X., Nie X., He C., Xiong X. (2011). Differential pathogenicity of two different recombinant PVY^NTN^ isolates in *Physalis floridana* is determined by the coat protein gene. Virol. J..

[21] Nie B., Singh M., Sullivan A., Singh R.P., Xie C., Nie X. (2011). Recognition and molecular discrimination of severe and mild PVY° variants of *Potato virus Y* in potatoes in New Brunswick, Canada. Plant Dis..

[22] Nie B., Singh M., Sullivan A., Murphy A., Xie C., Nie X. (2012). Response of potato cultivars to five isolates belonging to four strains of *Potato virus Y*. Plant Dis..

[23] Nie X., Singh R.P. (2003). Evolution of North American PVY^NTN^ strain Tu 660 from local PVY^N^ by mutation rather than recombination. Virus Genes.

[24] Chellappan P., Vanitharani R., Ogbe F., Fauquet CM. (2005). Effect of temperature on geminivirus-induced RNA silencing in plants. Plant Physiol..

[25] De Koeyer D., Nie X., Douglass K., Lagüe M., Gustafson V., Albert L., Singh M. Next generation sequencing for detection of viruses infecting potato. Proceedings of the 96th Annual Meeting of the Potato Association of America.

[26] Nolte P., Whitworth J.L., Thornton M.K., McIntosh C.S. (2004). Effect of seedborne *Potato virus Y* on performance of Russet Burbank, Russet Norkotah, and Shepody potato. Plant Dis..

[27] Carr J.P., Beachy R.N., Klessig D.F. (1989). Are the PR1 proteins of tobacco involved in genetically engineered resistance to TMV?. Virology.

[28] Nie X., Singh R.P., Tai G.C.C. (2002). Molecular characterization and expression analysis of 1-aminocylcopropane-1-carboxylate oxidase homologs from potato under abiotic and biotic stresses. Genome.

[29] Alamillo J.M., Saenz P., Garcia J.A. (2006). Salicylic acid-mediated and RNA-silencing defense mechanisms cooperate in the restriction of systemic spread of plum pox virus in tobacco. Plant J..

[30] Alvarado V., Scholthof H.B. (2009). Plant responses against invasive nucleic acids: RNA silencing and its suppression by plant viral pathogens. Semin. Cell Dev. Biol..

[31] Dunoyer P., Schott G., Himber C., Meyer D., Takeda A., Carrington J.C., Voinnet O. (2010). Small RNA duplexes function as mobile silencing signals between plant cells. Science.

[32] Mlotshwa S., Pruss G.J., Peragine A., Endres M.W., Li J., Chen X., Poethig R.S., Bowman L.H., Vance V. (2008). DICER-LIKE2 plays a primary role in transitive silencing of transgenes in Arabidopsis. PLoS One.

[33] Molnar A., Melnyk C.W., Bassett A., Hardcastle T.J., Dunn R., Baulcombe D.C. (2010). Small silencing RNAs in plants are mobile and direct epigenetic modification in recipient cells. Science.

[34] Szittya G., Molnár A., Silhavy D., Hornyik C., Burgyán J. (2002). Short defective interfering RNAs of tombusviruses are not targeted but trigger post-transcriptional gene silencing against their helper virus. Plant Cell.

[35] Ding S.W. (2010). RNA-based antiviral immunity. Nat. Rev. Immunol..

[36] Voinnet O. (2009). Origin, biogenesis, and activity of plant microRNAs. Cell.

[37] Schuck J., Gursinsky T., Pantaleo V., Burgyán J., Behrens S.E. (2013). AGO/RISC-mediated antiviral RNA silencing in a plant *in vitro* system. Nucleic Acids Res..

[38] Palauqui J.C., Elmayan T., Pollien J.M., Vaucheret H. (1997). Systemic acquired silencing: Transgene-specific post-transcriptional silencing is transmitted by grafting from silenced stocks to non-silenced scions. EMBO J..

[39] Donaire L., Wang Y., Gonzalez-Ibeas D., Mayer K.F., Aranda M.A., Llave C. (2009). Deep-sequencing of plant viral small RNAs reveals effective and widespread targeting of viral genomes. Virology.

[40] Llave C. (2010). Virus-derived small interfering RNAs at the core of plant–virus interactions. Trends Plant Sci..

[41] Qi X., Bao F.S., Xie Z. (2009). Small RNA deep sequencing reveals role for *Arabidopsis thaliana* RNA-dependent RNA polymerases in viral siRNA biogenesis. PLoS One.

[42] Wang X.B., Wu Q., Ito T., Cillo F., Li W.X., Chen X., Yu J.L., Ding S.W. (2010). RNAi-mediated viral immunity requires amplification of virus-derived siRNAs in Arabidopsis thaliana. Proc. Natl. Acad. Sci. USA.

[43] Wu Q., Luo Y., Lu R., Lau N., Lai E.C., Li W.X., Ding S.W. (2010). Virus discovery by deep sequencing and assembly of virus-derived small silencing RNAs. Proc. Natl. Acad. Sci. USA.

[44] Nie X., Singh R.P. (2002). A new approach for the simultaneous differentiation of biological and geographical strains of *Potato virus Y* by uniplex and multiplex RT-PCR. J. Virol. Methods.

[45] Nie X., Singh R.P. (2003). Specific differentiation of recombinant PVY^N:^° and PVY^NTN^ strains by multiplex RT-PCR. J. Virol. Methods.

[46] Mohapatra S.S., Poole R.J., Dhindsa R.S. (1987). Changes in protein patterns and translatable messenger RNA populations during cold acclimation of alfalfa. Plant Physiol..

[47] Sambrook J., Fritsch E.F., Maniatis T. (1989). Molecular cloning: A laboratory manual.

[48] Balme-Sinibaldi V., Tribodet M., Croizat F., Lefeuvre P., Kerlan C., Jacquot E. (2006). Improvement of *Potato virus Y* (PVY) detection and quantitation using PVY^N^-and PVY°-specific real-time RT-PCR assays. J. Virol. Methods.

[49] Nie X., Singh R.P. (2001). Differential accumulation of *Potato virus A* and expression of pathogenesis-related genes in resistant potato cv. Shepody upon graft inoculation. Phytopathology.

[50] Bookout A.L., Mangelsdorf D.J. (2003). Quantitative real-time PCR protocol for analysis of nuclear receptor signaling pathways. Nucl. Recept. Signal..

